# Preserved appendages in a Silurian binodicope: implications for the evolutionary history of ostracod crustaceans

**DOI:** 10.1098/rsbl.2024.0097

**Published:** 2024-05-22

**Authors:** David J. Siveter, Derek E. G. Briggs, Derek J. Siveter, Mark D. Sutton

**Affiliations:** ^1^ School of Geography, Geology and the Environment, University of Leicester, Leicester LE1 7RH, UK; ^2^ Department of Earth and Planetary Sciences, and Yale Peabody Museum, Yale University, New Haven, CT 06520-8109, USA; ^3^ Earth Collections, Oxford University Museum of Natural History, Oxford OX1 3PW, UK; ^4^ Department of Earth Sciences, University of Oxford, Oxford OX1 3AN, UK; ^5^ Department of Earth Sciences and Engineering, Imperial College London, London SW7 2BP, UK

**Keywords:** Binodicopina, Herefordshire Lagerstätte, Ostracoda, Podocopa, Silurian

## Abstract

Ostracod crustaceans originated at least 500 Ma ago. Their tiny bivalved shells represent the most species-abundant fossil arthropods, and ostracods are omnipresent in a wide array of freshwater and marine environments today and in the past. *Derima paparme* gen. et sp. nov. from the Herefordshire Silurian Lagerstätte (~430 Ma) in the Welsh Borderland, UK, is one of only a handful of exceptionally preserved ostracods (with soft parts as well as the shell) known from the Palaeozoic. A male specimen provides the first evidence of the appendages of Binodicopina, a major group of Palaeozoic ostracods comprising some 135 Ordovician to Permian genera. The appendage morphology of *D. paparme*, but not its shell, indicates that binodicopes belong to Podocopa. The discovery that the soft-part morphology of binodicopes allies them with podocopes affirms that using the shell alone is an unreliable basis for classifying certain fossil ostracods, and knowledge of soft-part morphology is critical for the task. Current assignment of many fossil ostracods to higher taxa, and therefore the evolutionary history of the group, may require reconsideration.

## 1. Introduction

Ostracod crustaceans are ubiquitous and profuse today, and their shells represent the most species-abundant arthropods in the fossil record. The group originated at least 500 Ma ago [1–4] and colonized all kinds of freshwater and marine environments [5,6]. Most ostracods, both living and fossil, are benthic/nektobenthic. Pelagic species (exclusively Myodocopa) originated with an ecological shift in the Silurian [7,8]. Fossil ostracods that preserve appendages are extremely rare [9]. There are only nine such species from the Palaeozoic (six Myodocopa, three Podocopa; some 20 specimens in total), five of which (all myodocopes) are from the Herefordshire Konservat-Lagerstätte (~430 Ma) in the Welsh Borderland, UK [10]. This Lagerstätte has furnished unrivalled testimony of the palaeobiology and phylogenetic affinities of a wide range of Silurian invertebrates, including many arthropod groups [11]. Here, we report a new exceptionally preserved species of ostracod from the Herefordshire fauna. Its shell morphology supports assignment to Binodicopina, a major group of Palaeozoic ostracods. Its preserved soft parts are the first known from a binodicope; they ally the species to the Podocopa and critically test the conventional shell-based taxonomy of ostracods and hence our understanding of the evolutionary history of the group.

## 2. Material and methods

Fossils of the Herefordshire Lagerstätte are preserved as calcitic in-fills in calcareous nodules within a volcaniclastic layer [12]. A virtual reconstruction of the single known specimen of the new ostracod was generated by serially grinding and photographing at 20 µm intervals [13], then removing extraneous material digitally and resolving fossil-matrix ambiguities using SPIERS software [14]. A final, colour-coded model was studied using interactive visualization, stereo-pairs, dissection and animation. The exact boundary between the body and limbs, as shown in the colour-coded illustrations, involves some interpretation.

## 3. Systematic palaeontology

The specimen (Oxford University Museum of Natural History, OUMNH PAL-C.36094: holotype) is classified as Euarthropoda, Crustacea, class Ostracoda, order Beyrichicopida [15], suborder Binodicopina [16], family Bolliidae [17], *Derima paparme* gen. et sp. nov., named for Carolyn Lewis (OUMNH), in recognition of her fundamental role in reconstructing many animals of the Herefordshire Lagerstätte: Greek, *deris*, battle + *ellogimos*, famous, from the Norman name *Lowis*, *Lodovicus*, after the latinized *Ludovicus* of the Germanic Hlūtwīg (‘famed battle’), which gave rise to the surname Lewis; *palaios*, ancient + *parme*, a shield used by foot-soldiers, alluding to the squat shell. Gender, feminine. Wenlock Series, Herefordshire, England.

### 3.1. Diagnosis

Bolliid with a squat-shaped postplete carapace with a broad lateroadmarginal bend and very wide admarginal surface between cardinal corners. Dorsally there is an anterior node and a weaker posterior lobal structure. There are seven limb pairs and a presumed furca bearing two well-developed lamellae.

### 3.2. Description

**Figure 1.**
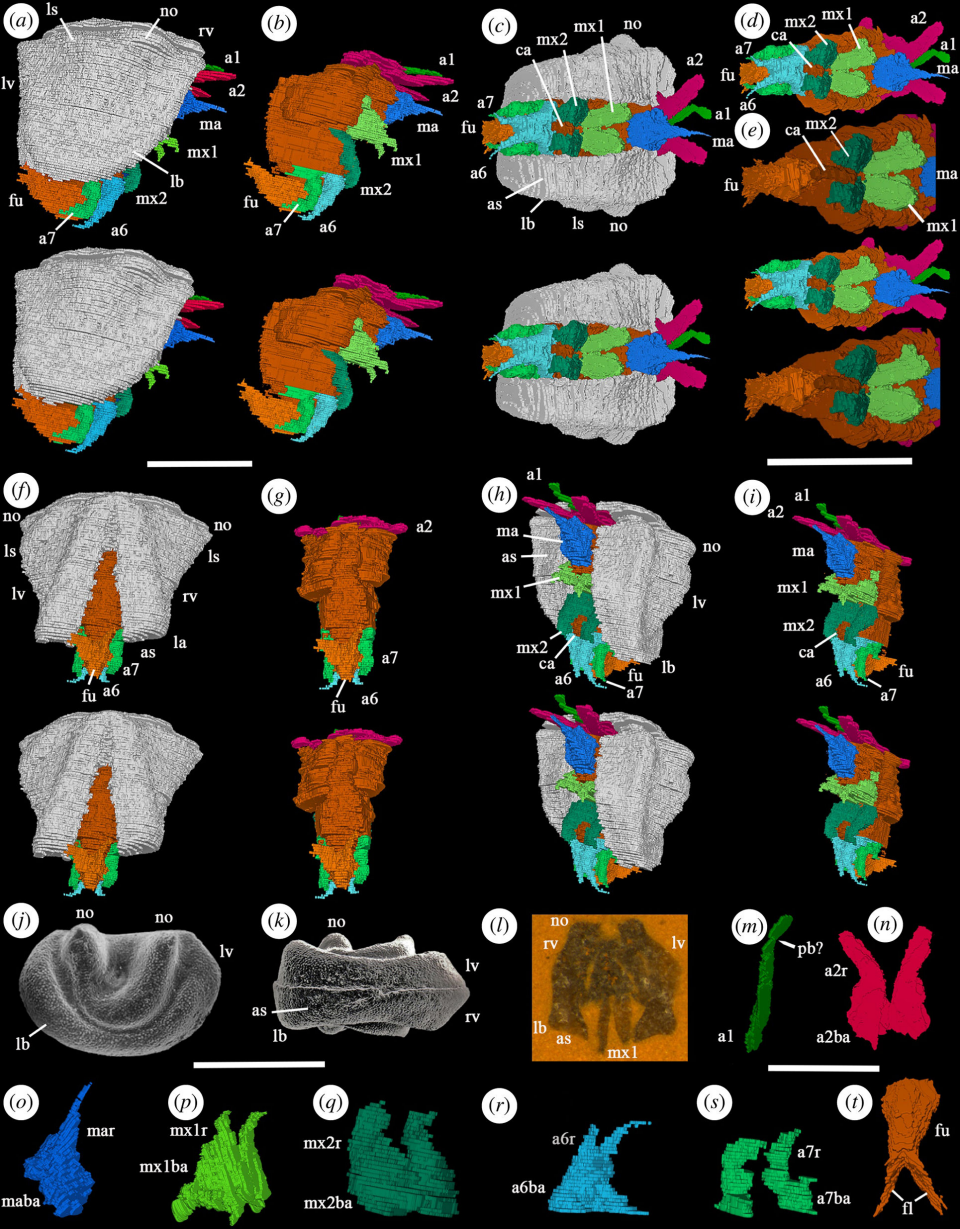
(***a–i,l–t***) *Derima paparme* (OUMNH PAL-C.36094): ‘virtual’ reconstructions (*a–i*: stereo-pairs). (***a,b***) Right lateral views; valves omitted in (***b***). (***c–e***) Ventral views; valves omitted in (***d***), valves and sixth and seventh pairs of limbs omitted in (***e***). (***f,g***) Posterior views; valves omitted in (***g***). (***h,i***) Anterior left lateral oblique views; valves omitted in (***i***). (***j,k***) *Bollia bicollina* [18] (type species of *Bollia*, the type genus of the Bolliidae), Buildwas Formation, Wenlock Series, Shropshire, UK: (***j***) left valve lateral view (Natural History Museum, London, OS 6638); (***k***) carapace, ventral view, anterior to the left (OS 6637). (***l***) Specimen in rock, viewed from anterior. (***m–t***) Appendages: (***m,o–s***) posterior left oblique views; (***n,t***) posterior views; (***m***) right first antenna; (***n***) second antenna pair; (***o***) mandible; (***p***) first maxilla pair; (***q***) second maxilla pair; (***r***) sixth limb pair; (***s***) seventh limb pair; (***t***) furca. All scale bars are 1.0 mm. Abbreviations: a1, first antenna; a2, second antenna; a2ba, limb base of the second antenna; a2r, ramus of the second antenna; a6, sixth limb; a6ba, limb base of the sixth limb; a6r, ramus of the sixth limb; a7, seventh limb; a7ba, limb base of the seventh limb; a7r, ramus of the seventh limb; as, admarginal surface; ca, copulatory appendage; fl, furcal lamellae; fu, furca; lb, lateroadmarginal bend; lv, left valve; ma, mandible; maba, limb base of mandible; mar, ramus of mandible; mx1, first maxilla; mx1ba, limb base of the first maxilla; mx1r, ramus of the first maxilla; mx2, second maxilla; mx2ba, limb base of the second maxilla; mx2r, ramus of the second maxilla; no, node; rv, right valve; pb?, podomere boundary?

The small size of the specimen relative to slice dimensions constrains the amount of detail that can be discerned. Carapace squat-shaped, postplete in lateral view (figure 1*a*). Maximum length (1.7 mm) between cardinal corners; maximum height (1.5 mm) just behind mid-length; maximum carapace width (*ca* 1.4 mm) above mid-height. Valves of the specimen gape at about 20° (figure 1*c,f*). The valve outline in lateral view is almost straight and vertical posteriorly (figure 1*a*), evenly and strongly curved ventrally, and forward sloping anteriorly ending in a small blunt forward projection at the anterior cardinal corner. A broad lateroadmarginal bend parallels a wide, shallow perilobal depression between the cardinal corners (figure 1*a*,*c*,*f,h*). The adjacent lateral valve area is gently tumid overall with the greatest inflation just above mid-height. An acuminate anterodorsal node anterior to a more weakly developed, slightly larger posterodorsal lobal structure occurs near (and extends slightly above?) the incompletely preserved dorsal valve margin (figure 1*a*,*f,h*). The admarginal surface of the valve is very wide between cardinal corners (figure 1*c*,*f,h*).

Seven pairs of limbs and a furca are evident, projecting beyond the carapace (figure 1*a*,*c*,*f,h*). Limb pairs 1–3 project forwards; pairs 4–7 and the furca are flexed gently overall towards the posterior. Possible evidence of podomere boundaries is only clearly discernible in the first appendage (figure 1*m*). Only a single ramus is evident in each appendage. A copulatory appendage is present. Internal organs are not preserved. Eyes are not evident.

Only the right appendage of the first antenna (antennule: figure 1*a*–*d*,*h*,*i,m*) is preserved. It originates close to the sagittal plane, projects anterolaterally as a long, evenly narrow ramus and is geniculate (podomere boundary?) at a point one-fifth of its length from the distal end. The second antenna (antenna: figure 1*a*–*d*,*g*–*i,n*) projects forward from a broadly elongate triangular structure (presumed basipod) and is wider but similar in length to the first. The morphology of the mandible (figure 1*a*–*d*,*h*,*i,o*) is difficult to discern. It is preserved as a broad-based structure from which projects an apparently single ramus slightly offset to the right of the sagittal line. The ramus is weakly curved and slenderly tapered to a point distally. A weak depression in the limb base to the left of the sagittal line may represent the site of a missing (left) appendage. Alternatively, the broad basal area and the ramus may represent the left and right appendages preserved fused together; or the broad basal area may represent fused left and right appendages together with only one (the right) ramus preserved. It is not possible to determine whether the ramus represents an exopod or endopod (this is also the case for the ramus of limbs 2, 4–7).

Limbs 4–7 are approximately equal in size. The limb base of the first maxilla (maxillula: figure 1*a*–*e*,*h*,*i*,*l,p*) is a broad triangular structure presumably representing a basipod and possibly a proximal endite. Distally it bears a short, gently tapered ramus. The edges of the opposing basipods touch sagitally at the presumed site of the atrium oris, but finer morphological details cannot be discerned. The second maxilla (fifth limb: figure 1*a*–*d*,*h*,*i,q*) arises immediately behind the first. Its broad limb bases meet sagitally; each bears a short, stout, tapered ramus. The sixth limb (figure 1*a*–*i,r*) is similar to the fourth, comprising a broad limb base (presumed basipod), the inner edges of which pair meet sagitally, and a short, slender, tapered ramus that terminates at a point. The seventh limb (figure 1*a*–*i,s*) has a smaller limb base, the inner edges of which do not meet sagitally, and a stout, pointed ramus. The furca is well developed (figure 1*a*–*d*,*f*–*i,t*). The base is preserved partly enveloped by the sixth and seventh limb pairs (figure 1*b*–*d*,*h,i*). It bears a pair of long, prominent furcal lamellae that taper posteriorly. Furcal claws are not evident though this may represent a taphonomic loss. A well-developed digitate feature projects forward from the furca to between the fifth and sixth limb pairs (figure 1*c*–*e*,*h,i*). Its size, shape and position identify it as a copulatory appendage, and the specimen is interpreted as a male. There is no evidence that the copulatory appendage represents hemipenes, but this may be a preservational factor.

## 4. Discussion

Based on shell morphology, *D. paparme* is assigned to the Bolliidae within Binodicopina. Binodicopes comprise some 135 Ordovician to Permian genera (David Siveter 2023, unpublished data). Representatives are common in the Ordovician and Silurian (e.g. [19–22]). As exemplified by *Bollia bicollina* (figure 1*j,k*), the type species of the type genus, bolliids display a relatively simple valve morphology bearing 1–3 dorsal nodes which in some cases are connected by a ridge or ridges; a rounded lateroadmarginal bend ([19]: ‘pseudovelum’); and a wide, simple admarginal surface. Details of the lobal structures and lateroadmarginal bend of *D. paparme* together distinguish the shell from all other bolliids. It lacks a well-defined lateral lobal connection as present, for example, in *Bollia* and *Ullehmannia* [23]. The presence of a lateroadmarginal bend distinguishes it from genera such as the binodal *Klimphores* [24], and its lateroadmarginal bend is much narrower and more strongly curved than in, for example, the three-lobed *Bullaeferum* [25]. The lobal structures of *D. paparme* are much weaker and shorter than in some other binodicopes such as *Kimsella* [26].

All three major groups (subclasses) of ostracod—Myodocopa, Podocopa and Palaeocopa—occur in the Palaeozoic. Myodocopa and Podocopa have been resolved as clades based on soft-part morphological and molecular analyses [1,2,27]. Palaeocopes are known from the Palaeozoic and, extremely rarely (e.g. [28]), from the Triassic. They are represented by tens of thousands of described species based on their shells alone; their appendages are unknown [29]. Based on shell characteristics, the Binodicopina have been included within Palaeocopa (e.g. [20,30–32]), but others place binodicopes and palaeocopes as separate suborders within order Beyrichicopida ranked alongside Podocopa (e.g. [19,21,22,33,34]).

Given the well-developed copulatory appendage, the morphology of the *D. paparme* specimen indicates a mature individual rather than a pre-adult stage. Aspects of its appendage morphology, the first known evidence of the limbs of binodicopes, are incompatible with an assignment to the Myodocopa. Most obviously, the seventh appendage is unlike that in myodocopid myodocopes in being ‘leg-like’ rather than vermiform and is unlike that of halocyprid myodocopes in being well developed rather than reduced or absent (see [35] for illustrations of podocope and myodocope soft parts). In addition, the basipod of the second antenna is not a large, rounded/almond-shaped structure, and there are no large epipods on the fifth or sixth limb as is characteristic of myodocopids and some halocyprids [35]. Other soft-part characters that distinguish podocopes from myodocopes, such as having the anus behind the furca and lacking a bellonci sensory organ on the head [35], are not evident/detectable in *D. paparme*. The absence of a second ramus in the limbs of the *D. paparme* specimen may reflect partial or complete reduction (in many ostracods it is represented by a seta(e)) and/or it may not be preserved or technically recoverable in such a small specimen.

In contrast, the number, position and general morphology of the appendages of *D. paparme,* especially the well-developed leg-like seventh limb, support an assignment of the species and, *ipso facto,* the Bolliidae and Binodicopina to Podocopa. Classifications that place Binodicopina within Palaeocopa (see above) would potentially implicate thousands of additional (palaeocope) genera as podocopes.

The shell and appendage morphology of *D. paparme* show no specialization for a pelagic lifestyle. In contrast to pelagic myodocopes [7,8], it lacks a rostrum and rostral incisure and an array of ‘natatory’ setae and a large basipod on the second antenna. It was, like all podocopes, probably benthic/nektobenthic.

The discovery that their appendage morphology places binodicopes with podocopes confirms the critical role that soft parts play in determining the affinities of especially Palaeozoic ostracods. Current high-level ostracod classifications and the placement of many hundreds (possibly thousands) of fossil ostracod species in higher taxa may be suspect. The shape and lobal/lateroadmarginal morphology of the carapace of *D. paparme* echo some aspects of palaeocope valves but is unlike that of any known living or fossil podocope including the extant puncioids which are atypical in having eight limb pairs. The only known Palaeozoic podocopes with preserved appendages—the Devonian *Cytherellina submagma* [36], the Carboniferous *Palaeocypris edwardsii* [37] and Podocopida indet [9]—have the simple bean-like shell shape characteristic of podocopes. The five other Palaeozoic ostracod species with preserved appendages are definitively myodocope, but their shell morphology shows high diversity, from the typical myodocope-like *Nasunaris flata* [38] to the more palaeocope-like *Pauline avibella* [39]. The binodicope *D. paparme* adds another major ostracod group to the evidence demonstrating the importance of exceptional preservation to interpretations of the evolutionary history of the Ostracoda.

## Data Availability

The model (VAXML/STL format) plus the datasets from the serial grinding of OUMNH PAL-C.36094 are housed at OUMNH and Dryad [40].
